# Hydralazine Protects Nigrostriatal Dopaminergic Neurons From MPP^+^ and MPTP Induced Neurotoxicity: Roles of Nrf2-ARE Signaling Pathway

**DOI:** 10.3389/fneur.2019.00271

**Published:** 2019-03-20

**Authors:** Xingfang Guo, Chao Han, Kai Ma, Yun Xia, Fang Wan, Sijia Yin, Liang Kou, Yadi Sun, Jiawei Wu, Junjie Hu, Jinsha Huang, Nian Xiong, Tao Wang

**Affiliations:** ^1^Department of Neurology, Union Hospital, Tongji Medical College, Huazhong University of Science and Technology, Wuhan, China; ^2^Department of Neurology, The First Affiliated Hospital of USTC and Division of Life Sciences and Medicine, University of Science and Technology of China, Hefei, China

**Keywords:** Parkinson's disease, hydralazine, neuroprotection, Nrf2-ARE signaling pathway, MPTP, MPP^+^

## Abstract

Although the pathogenic mechanisms of Parkinson's disease (PD) remain unclear, ample empirical evidence suggests that oxidative stress is involved in the pathogenesis of this disease. The nuclear factor E2-related factor 2 (Nrf2) is known to activate several antioxidant response element (ARE)-driven antioxidative genes that prevents oxidative stress *in vitro* and *in vivo*. Moreover, it was documented that hydralazine is a potent Nrf2 activator. In this study, we tested whether hydralazine can attenuate 1-Methyl-4-phenylpyridinium (MPP^+^) and 1-methyl-4-phenyl-1,2,3,6-tetrahydropyridine (MPTP)- induced neurotoxicity *in vitro* and *in vivo* by activating Nrf2 and its downstream network of antioxidative genes. We found that treatment with hydralazine attenuated MPP^+^ or H_2_O_2_-induced loss of cell viability in human neuroblastoma cell line (SH-SY5Y). In addition, hydralazine significantly promoted the nuclear translocation of Nrf2, and upregulated the expression of its downstream antioxidative genes. Further, knockout of Nrf2 abolished the protection conferred by hydralazine on MPP^+^ -induced cell death. Similar findings were observed *in vivo*. Before, during, and after MPTP 30 mg/kg (i.p.) administration for 7 days, the mice were given hydralazine (Hyd) 51.7 mg/kg per day by oral gavage for 3 weeks. Oral administration of hydralazine ameliorated oxidative stress, MPTP-induced behavioral disorder, and loss of neurons of dopaminergic system in the substantia nigra (SN) and striatum, all of which were attributed to its ability to activate the Nrf2-ARE pathway. Hydralazine increased the migration of Nrf2 to the nucleus in dopaminergic neurons, enhanced the expression of its downstream antioxidative genes. Together, these datasets show that the Nrf2-ARE pathway mediates the protective effects of hydralazine on Parkinson's disease.

## Highlights

- Hydralazine prevents the MPP^+^ and H_2_O_2_-induced cell death in SH-SY5Y cells, and activates Nrf2-ARE signaling in both treated and non-treated MPP ^+^ cells *in vitro*.
- Hydralazine activate Nrf2-ARE signaling and attenuate MPP^+^-mediated cytotoxicity in an Nrf2-dependent pattern.
- Hydralazine confers protection in dopaminergic neurons in the MPTP model of Parkinson's disease.
- Hydralazine alleviate oxidative stress and activates Nrf2-triggered gene expression *in vivo*.

## Introduction

Currently, the pathogenesis of Parkinson's disease (PD) is elusive. It has been postulated that prolonged increased in reactive oxygen species (ROS) plays important role in modulating the occurrence of the disease (1, 2). This is because ROS compromises the mechanisms that balances oxidant and antioxidant systems (3). One of the mechanisms that prevent cell damage caused by oxidative stress is the antioxidant defense system. By increasing the level of antioxidant enzymes, phase II detoxifying enzymes, quinone oxidoreductase 1 (NQO1), NAD(P)H, heme oxygenase-1 (HMOX1), glutamate-cysteine ligase subunits (GCLC and GCLM), the nuclear factor E2-related factor 2 (Nrf2) modulates the pathophysiological and physiological processes of various diseases (4). These enzymes are involved in glutathione (GSH) synthesis and maintains the form of GSH by suppressing its oxidized form GSSG (5, 6). Several structural and molecular studies on Nrf2 revealed that Nrf2 is expressed in the cytoplasm in physiological conditions via a Kelch-like ECH-associated protein 1 (Keap1)-dependent ubiquitination-proteasomal degradation, and this process is enhanced by electrophiles and oxidants (7, 8). It was revealed that the N terminal region and double glycine repeat domain (DGR) of Keap1 contains the bric-a-brac, tramtrack, broad-complex (BTB) domain, termed as Kelch repeats in the C terminal region. Keap1 keeps the Nrf2 within the cytoplasm in normal cellular conditions by binding to a scaffold protein of Nrf2 ubiquitin ligase (E3), Cul3 through its BTB domain, and binding to the substrate of Nrf2 through its DGR domain, which causes Nrf2 degradation (9) and ubiquitination (10). When stimulated by oxidants or electrophiles, Nrf2 undergoes modifications by some mechanisms which compromise Keap1/Nrf2 interactions, which leads to the dissociation of Nrf2 from Keap1 complex, become stabilized, and then moves into the nucleus. This is followed by binding to the antioxidant response elements (AREs), a consensus gene sequence located in the promoter region of several genes that encode antioxidant enzymes (11). Several studies have provided evidence that Nrf2-ARE signal transduction participates in PD (12, 13). Downregulation of Nrf2 renders the dopaminergic neurons susceptible to oxidative stress damage (14–16), while activation of Nrf2 confers neuroprotection (17–21). Based on this background, the Nrf2-ARE interaction is thought to play important role in neurodegenerative conditions including PD.

Administration of pharmacological antioxidants seem to be the most direct approach to suppress oxidative damage. However, clinical trials on antioxidants e.g., N-acetylcysteine, vitamin E, glutathione, and vitamin C have revealed rather disappointing results for PD (22–25). One possible reason is that the efficacy of these antioxidants is largely based on their ability to stoichiometrically scavenge for oxidants. An alternative promising therapeutic strategy of restoring redox homeostasis in PD by activating the transcription factor Nrf2 may have significant advantages over conventional strategies. Hydralazine (Hyd), an FDA approved treatment for hypertension, is a water-soluble carbonyl-scavenger due to its nucleophilic hydrazine group (26). Because of this, hydralazine has been found to be a powerful antioxidant properties (27–29), Moreover, it was reported that hydralazine could activate the Nrf2 signaling pathway *in vitro* [human neuroblastoma cell line (SH-SY5Y)] and *in vivo* (Caenorhabditis elegans) model system (30), and possesses anti-aging properties (31). As mentioned above, oxidative stress and the deregulation of Nrf2-ARE signaling pathways are both involved in PD. Thus, we hypothesized that hydralazine may provide strong neuroprotective effects in Parkinson's disease in both *in vitro* and *in vivo* settings. To test this possibility, we investigated the mechanisms that orchestrate the neuroprotective effects of hydralazine using an 1-Methyl-4-phenylpyridinium (MPP^+^)-induced cytotoxicity model and 1-methyl-4-phenyl-1,2,3,6-tetrahydropyridine (MPTP)-induced mice model of PD. We found that hydralazine displayed promising therapeutic efficacy toward PD by activating the Nrf2 signaling pathway.

## Experimental Procedures

### Preparation of Human Neuroblastoma SH-SY5Y Cells

SHSY5Y cells were obtained from ATCC (ATCCCRL-2266) and grown in DMEM/F12 medium (hyclone) supplemented with 10% FBS (EVERY GREEN, Zhejiang Tianhang Biotechnology Co., Ltd, China), 100 μg/mL streptomycin, and 100 U/ml penicillin (Beijing solarbio science & technology co., Ltd) in high humidity condition with 5% CO_2_ at 37°C. After culturing the cells in 100 mm dishes to reach a ~70% confluence, they were subjected to hydralazine, H_2_O_2_ or MPP^+^ treatment. The dose and duration of application of hydralazine, H_2_O_2_ or MPP^+^ are provided in the figures and text. SiRNA interference were performed by treating the cells with Nrf2 SiRNA (sc-37030) or control SiRNA (sc-37007) (Santa Cruz Biotechnology, Santa Cruz, CA) in 6-well plates for 24 h using the Lipofectamine 3000 reagent (Thermo Fisher Scientific Co., Carlsbad, CA, USA) as indicated in the instructions provided by the manufacturer. After transfection for approximately 24 h, SHSY5Y cells were exposed to hydralazine with or without MPP^+^. After these treatments, cells were used for biochemical analysis.

### Cell Viability Evaluation by CCK-8 Assay

The cell counting kit-8 solution (CCK-8) assay was performed to determine the cell viability. Briefly, after seeding the SH-SY5Y cells in 96-well plates at a density of 1 × 10^4^ cells/well, they were treated with reagents. This was followed by incubating with 10 μl CCK-8 buffer for 1 h at 37 C following the instructions provided by the kit company. A microplate reader (BioTek, Winooski, VT, USA) was used to measure the absorbance at 450 nm. All samples were assessed in triplicate.

### Quantitative Real-Time PCR

Total RNA was isolated from SHY-SY5 cells using RNAiso Plus (TaKaRa, Japan). Total RNA (2 μg) was reverse transcribed to cDNA using the PrimeScript™ II 1st Strand cDNA Synthesis Kit (TaKaRa, Japan) to determine the mRNA expressions of Nrf2 by qRT-PCR using SYBR Green reagent (TaKaRa, Japan). The PCR condition was as follows: 95°C for 5 min, 60°C for 20 s, 40 amplification cycles. Housekeeping gene β-actin served as an internal control. Data analysis is based on the ΔΔCt method with normalization of raw data to β-actin. Each reaction was run in triplicate. Nrf2 primer: forward, 5′-CAGTCAGCGACGGAAAGAGT-3′; reverse, 5′-ACGTAGCCGAAGAAACCTCA-3′; β-actin primer: forward, 5′- AGCCATGTA CGTAGCCATCC−3′; reverse, 5′- CTCTCAGCTGTGGTGGTGAA -3′.

### Animals and Treatment

The mice used in this study were kept and handled according to the guidelines of the NIH Guide regarding the Use and Care of Laboratory Animals. All animals were given free water and food *ad libitum*, and the housing conditions were regulated to a 12 h dark-light cycle and temperature of (22 ± 2°C). This study conformed to the guidelines of the Animal Care and Use Committees of Maximum efforts of Huazhong University of Science and Technology (HUST). Care was taken to use few animals and reduce discomfort. Male C57/BL6 mice, 8 weeks old, were used all of which were obtained from Beijing Vital River Laboratory Animal Technology Co., Ltd. There are four groups (*n* = 8 or 9 for per group) in this experiment. The first group mice (MPTP group) only received injections of MPTP-HCl (30 mg/kg, i.p., Sigma) in saline for consecutive 7 days, an MPTP model of PD was generated as previously described (32). The second group mice (Hyd+MPTP group) were administered hydralazine (51.7 mg/kg per day in saline, Sigma) (33) by oral gavage for 3 weeks before, during, and after MPTP administration. The third group mice (Hyd group) were administered hydralazine (51.7 mg/kg per day in saline, Sigma) (33) by oral gavage for 3 weeks, and the fourth group (Control group) received vehicle only. Behavioral test of the animals were performed after the last oral gavage, after behavior test, mice were killed (Figure 3A).

### Rotarod Testing

The general motor deficits were evaluated using the rotarod test. Mice were put in a rod which was 7 cm in diameter, and then tested at a constant speed of 30 rpm. In each test, the mouse was subjected to the rod for 1 min prior to the test. Each animal was put on the roller for 5 min, and then the latency to fall off the rolling rod was calculated. Prior to assessing the behavior of the animals, they were given pre-trials for habituation to the test system for 4 days. An average of five trials were performed for each mouse.

### Pole Testing

Each mouse was placed on the top of a vertical pole (50 cm long and 1 cm in diameter) wrapped with gauze to avoid slip and fall. After pre-trials for habituation to the test system for 4 days, time of the mouse head orient downward (named as Turn) and total time climbing down the pole as an indication of the locomotion activity (TLA) were recorded. Each mouse received five successive trials for average.

### Tissue Preparation

One day after the behavior test, animals were killed by decapitation under anesthesia. Afterwards, brains were removed immediately was fixed in 4% buffered paraformaldehyde and embedded in paraffin for the next immunofluorescent staining. The ventral midbrain containing the substantia nigra and striatum of the other mice were dissected and frozen immediately in liquid nitrogen and stored at −80°C for protein extraction.

### Immunofluorescent Staining

After isolation, the substantia nigra and striatum tissues were embedded in paraffin and sectioned to 5 μm thickness. The sections were deparaffinized with xylene, followed by dehydration using ethanol. Finally, EDTA (PH 9.0) antigen retrieval buffer was used for antigen retrieval. These section were put on slices followed by treatment with 3% BSA at room temperature for 30 min, before they were incubated with the following primary antibodies at 4°C overnight, mouse polyclonal anti-TH (1:200, Proteintech, China), rabbit polyclonal anti-Nrf2 (1:200, Genetex), rabbit monoclonal anti-IBA1(1:200, Abcam), rabbit monoclonal anti-GFAP (1:200, Abcam). Subsequently, secondary antibodies, Alexa Fluor 488-conjugated (1:500, Life Technologies) and/or Alexa Fluor 647-conjugated (1:500, Life Technologies) were added and incubated for 1 h at room temperature. SH-SY5Y cell were fixed with 4% paraformaldehyde for 30 min, washed in PBS, and permeabilized with 0.3% Triton X-100 in PBS for 10 min at room temperature. The cells were treated with rabbit polyclonal anti-Nrf2 (1:200, Genetex) antibody overnight at 4°C after blocking with 10% normal goat serum for 1 h, and then incubated with Alexa Fluor 488-conjugated (1:500, Life Technologies) secondary antibody for 1 h at room temperature. Images from the sections were examined on an OLYMPUS IX71 fluorescent microscope. Then quantitative analysis of the optical density and of number positive neurons was performed using Image pro plus.

### Determination of Protein Expression

Nuclear and cytosolic fractions were extracted from the substantia nigra, striatum specimen and human neuroblastoma SH-SY5Y cells using cytoplasmic and nuclear protein extraction kit (Beyotime, Beijing, China) following the instructions given by the manufacturer. Briefly, cells were subjected to various treatments and then suspended in PBS. The next step involved the addition of the cytoplasmic protein isolation reagents A and B. The cytosolic fraction was obtained by centrifugation. The nuclei pellets were suspended in the isolation reagent for nuclear protein. Similarly, the nuclear fraction was obtained by centrifugation. All the isolated proteins from mouse striatum, ventral, and SH-SY5Y cells were treated on ice with RIPA lysis buffer comprising of phosphatase inhibitor A and B, protease inhibitor PMSF (PMSF:RIPA = 1:99), cocktail (Servicebio, Wuhan, China), followed by centrifugation at 12,000 g, for 15 min at 4°C, to obtain the supernatants. BCA Protein Assay Kit was applied to measure the protein concentration. This was followed by denaturation of the protein specimen using the sodium dodecyl sulfate solution together with boiling for 5 min at 98°C. About 30 μg sample was loaded onto SDS-PAGE after which electrophoresis was performed to transfer the proteins to a PVDF membrane. Thereafter, 5% milk was using to block the membranes at room temperature for 1 h before primary antibodies, rabbit monoclonal anti-β-actin (1:3000, Antgene, China), rabbit polyclonal anti-Histone H3 (1:1000, Servicebio, China), mouse monoclonal anti-GCLC (1:500, Santa Cruz), rabbit polyclonal anti-Nrf2 (1:1000, Genetex), rabbit monoclonal anti-NQO1 (1:10000, Abcam), rabbit monoclonal anti-HMOX1 (1:2000, Abcam), rabbit monoclonal anti-GCLM (1:5000, Abcam), and rabbit polyclonal tyrosine hydroxylase antibody (1:1000, Proteintech, China) were added and incubated overnight at 4°C. Finally, HRP-conjugated secondary anti-mouse antibody (1:5000, Antgene, China) or HRP-conjugated secondary anti-rabbit (1:5000, Antgene, China) was added to the membranes after washing with TBST thrice (5 min/each time) for 1 h at room temperature. After washing, ECL was loaded onto the membrane to detect the immunoreaction using the Bio-Rad imaging system. The branes were analyzed with Imagine J software. Relative band intensities were measured with β-actin or Histone H3 serving as internal control.

### Determination of the Oxidative Activity by Lipid Peroxidation Assay

The generation of malondialdehyde (MDA) was measured using the lipid peroxidation commercial assay kit following the instructions provided by the manufacturer. Briefly, and tissue were homogenized, sonicated, and centrifuged. The MDA concentration on in the supernatants was measured as well as the protein concentration. The protein concentration was determined using the assay kit (Bio-Rad Laboratories, Hercules, USA). This was followed by performance of the MDA assay following the instructions provided with the Lipid Peroxidation MDA assay kit (Beyotime). The multimode microplate reader was used to quantify the MDA levels in the samples at 532 nm.

### Determination of Cellular Levels of GSSG and GSH Using HPLC

Substantia nigra samples were homogenized, sonicated and centrifuged. In brief, 30 μl supernatant was isocratically eluted through a 4.6 × 150 mm C18 column (ESA, Inc.) with a mobile phase containing 50 mM LiH_2_PO_4_, 1.0 mM 1-octanesulfonic acid, and 1.5% (v/v) methanol, and then detected by a 2-channel Coulochem III electrochemical detector (ESA, Inc), set with a guard cell potential 950 mV, Channel 1 potential for GSH detection and Channel 2 potential 880 mV for GSSG detection (34). The quantities of GSSG and GSH were presented as nmol per mg protein.

### Data Analysis

All data are expressed as mean ± SEM and were performed using the SPSS20 software. Between-group comparisons were conducted by Student's *t*-test, while the one-way ANOVAs was used to compare three or more groups followed by Tukey's multiple comparison test. A *P*-value of < 0.05 was considered to be statistically significant.

## Results

### Hydralazine Prevents the MPP^+^ and H_2_O_2_-Induced Cell Death in SH-SY5Y Cells, and Activates Nrf2-ARE Signaling in Both Treated and Non-treated MPP ^+^ Cells *in vitro*

**Figure 1:**
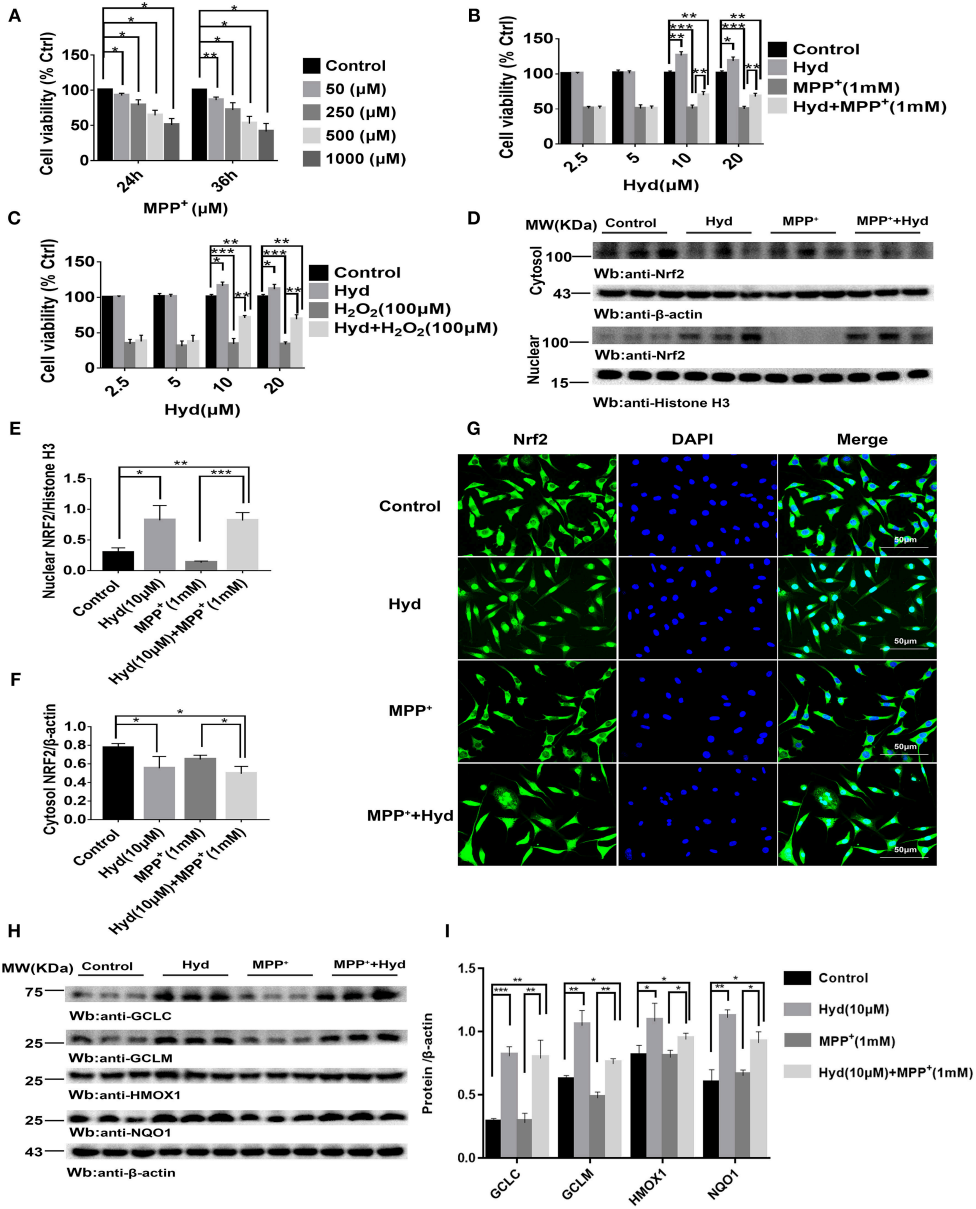
Hydralazine prevents the MPP^+^ and H_2_O_2_-induced cell death in SH-SY5Y cells, and activates Nrf2-ARE signaling in both treated and non-treated MPP ^+^ cells *in vitro*. SH-SY5Y cells were incubated with various concentrations of MPP^+^ for 24, 36 h **(A)**. Cell viability was determined by CCK-8 assay. ^*^p < 0.05, ^**^p < 0.01, *n* = 6, compared with the two indicated groups. SH-SY5Y cells were incubated with or without hydralazine (2.5, 5, 10, 20 μM) in the absence or presence of 1 mM MPP^+^ for 24 h **(B)**. The viability of cells under MPP^+^-mediated cytotoxicity was significantly improved with hydralazine (10 and 20 μM) treatment. ^*^p < 0.05, ^**^p < 0.01, ^***^p < 0.001, *n* = 6, significant difference between the two indicated groups. SH-SY5Y cells were incubated with or without hydralazine (2.5, 5, 10, 20 μM) in the absence or presence of H_2_O_2_ (100 μM) for 24 h **(C)**. The viability of cells under H_2_O_2_ -induced cytotoxicity was significantly improved with hydralazine (10 and 20 μM) treatment. ^*^p < 0.05, ^**^p < 0.01, ^***^p < 0.001 *n* = 6, significant difference between the two indicated groups. The results of immunofluorescence and western blot analysis indicated Nrf2 translocated to the nucleus with hydralazine treatment when exposured to MPP^+^ or not in SH-SY5Y **(D–G)**. Scale bar = 500 μm. Treated cells were subjected to cell fractionation and western blot analysis. β-actin or Histonen H3 served as a loading control. ^*^p < 0.05, ^**^p < 0.01, ^***^p < 0.001, *n* = 6, significant difference between the two indicated groups. Hydralazine induces expression of Nrf2-dependent antioxidant enzymes measured by western blot analysis **(H,I)**. ^*^p < 0.05, ^**^p < 0.01, ^***^p < 0.001, *n* = 6, significant difference between the two indicated groups. Data were presented as mean ± SEM.

Analysis of the influence of MPP^+^ on SH-SY5Y viability revealed that the cell viability decreased to about 50% or below based on CCK-8 assays when exposed to 1000 μM (1 mM) MPP^+^ for 24 or 36 h (Figure 1A). Therefore, we treated SH-SY5Y cells with MPP^+^1 mM for 24 h to establish a model of MPP^+^-induced cytotoxicity. Next, the effects of hydralazine on the survival of cells following exposure to MPP^+^ were tested. Four doses of hydralazine (2.5, 5, 10, or 20 μM) were tested against MPP^+^. Hydralazine alone at the micromolar range (2.5–20 μM) had no overt effects on these cells. But, hydralazine (10 and 20 μM) conferred significant protection against MPP^+^ according to the CCK-8 assays (Figure 1B). Moreover, we chose H_2_O_2_ (100 μM) as another control of toxicity (30). Similarly, hydralazine protected the cells from H_2_O_2_-induced cytotoxicity (Figure 1C). Moreover, we observed that hydralazine (10 and 20 μM) alone increased the viability of SH-SY5Y in the absence of MPP^+^ and H_2_O_2_ (Figures 1B,C). It is well-known that during cellular metabolism, spontaneous oxidative damage to unsaturated lipids generates many electrophilic carbonyl compounds which are potential threats to cell survival. Since hydralazine is a well-known carbonyl scavenger (35), we speculated that it can improve SH-SY5Y viability by scavenging Carbonyl moieties.

Thereafter, we tested the ability of hydralazine to induce nuclear translocation of Nrf2. SH-SY5Y cells were treated with 10 μM hydralazine with or without MPP^+^, but the control groups were treated with saline following Nrf2 partition quantification and subcellular fractionation. Hydralazine treatment increased the nuclear translocation of Nrf2, which was accompanied by a corresponding decreased in the cytosolic fraction relative to the nuclear Nrf2 fraction (Figures 1D–F). The same phenomenon of nuclear translocation was observed based on immunofluorescence (Figure 1G). As showed in Figures 1D–G, treatment with hydralazine alone triggered a remarkable increase in the nuclear translocation of Nrf2, but the reasons for this effect remain unclear. It is likely that Nrf2 was modified by hydralazine in a way that disrupted the Nrf2:Keap1 interaction, and then, translocated to the nucleus. Further studies are required to answer this question. Next, we determined whether hydralazine (10 μM) would increase the downstream protein of the Nrf2-ARE pathway, GCLC, GCLM, HMOX1, and NQO1 by western blot analysis. All four protein levels of target genes were significantly increased (Figures 1H,I). Collectively, the *in vitro* findings indicate that hydralazine induce nuclear translocation of Nrf2 which activates the ARE genes, thereby conferring protection against MPP^+^.

### Hydralazine Activate Nrf2-ARE Signaling and Attenuate MPP^+^-Mediated Cytotoxicity in an Nrf2-Dependent Pattern

**Figure 2:**
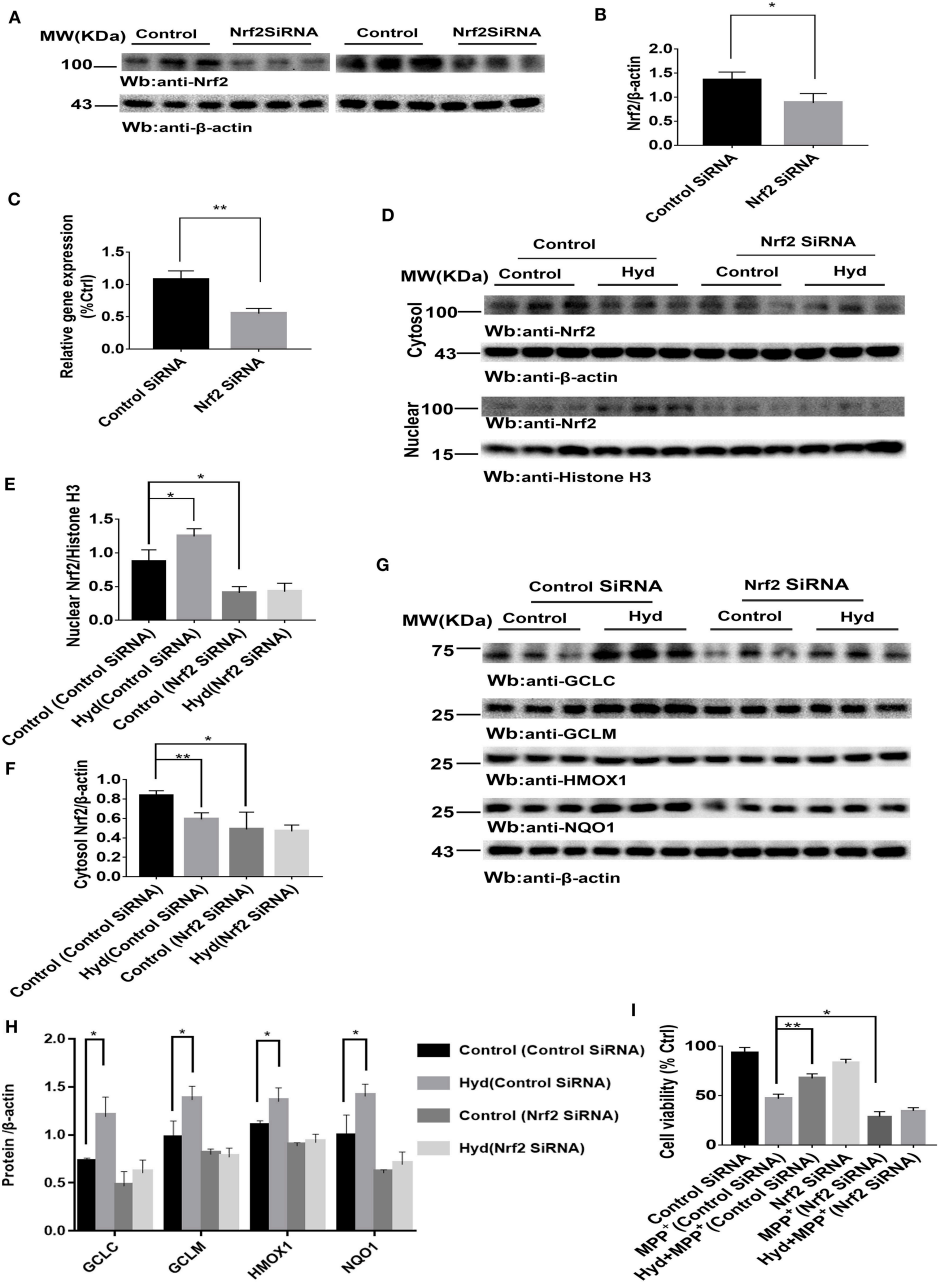
Hydralazine activate Nrf2-ARE signaling and attenuate MPP^+^-mediated cytotoxicity in an Nrf2-dependent pattern. SH-SY5Y cells were treated with either an Nrf2 SiRNA or a control SiRNA. The protein and mRNA levels of Nrf2 were measured by western blotting and real-time PCR **(A–C)**. ^*^p < 0.05, ^**^p < 0.01, *n* = 6, significant difference between the two indicated groups. Hydralazine treatment alone induced a remarkable increase in the nuclear translocation of Nrf2, however, Nrf2 interference abolished the increase in nuclear Nrf2 induced by hydralazine in SH-SY5Y **(D,E)**, in the meantime, cytosolic Nrf2 had corresponding decrease in contrast with control group transfected with control SiRNA **(D,F)** by western blot analysis. ^*^p < 0.05, ^**^p < 0.01, *n* = 6, significant difference between the two indicated groups. Selective activation of Nrf2 mediated gene transcription by hydralazine in SH-SY5Y cells transfected by control SiRNA, but not in SH-SY5Y cells transfected by Nrf2 SiRNA measured by western blotting **(G,H)**. ^*^p < 0.05, *n* = 6, significant difference between the two indicated groups. Hydralazine significantly protected SH-SY5Y cells from MPP^+^ induced death in an Nrf2-dependent manner measured by CCK-8 assay **(I)**. ^*^p < 0.05, ^**^p < 0.01, *n* = 6, significant difference between the two indicated groups. Data were presented as mean ± SEM.

To confirm whether the Nrf2-dependent pathway mediated the neuroprotective effects of hydralazine, the inhibitory effect of hydralazine on MPP^+^ neurotoxicity was assessed in SH-SY5Y cells after transfection with either control SiRNA or Nrf2 SiRNA. Results from western blotting and real-time PCR indicated that Nrf2 was successfully knocked-down in SH-SY5Y cells by Nrf2 SiRNA compared to control SiRNA (Figures 2A–C). Treatment with hydralazine alone triggered a remarkable increase in the nuclear translocation of Nrf2 (Figures 2D–F). However, Nrf2 interference abolished the increase in nuclear Nrf2 caused by hydralazine in SH-SY5Y cells. On the other hand, cytosolic Nrf2 displayed a corresponding decrease relative to the control group transfected with control SiRNA. Furthermore, the hydralazine-induced increase in expression of Nrf2-mediated ARE genes, such as GCLC, GCLM, HMOX1, and NQO1 were inhibited following Nrf2 SiRNA in SH-SY5Y cells based on western blot analysis (Figures 2G,H). According to these results, we speculate hydralazine activate Nrf2-ARE signaling in an Nrf2-dependent manner.

Meanwhile, our results indicated that hydralazine treatment increased cell viability, which is opposite to the CCK8 assay results recorded in MPP^+^ exposure alone group in SH-SY5Y cells transfected with control SiRNA (Figure 2I, *p* < 0.01). However, hydralazine failed to increase cell viability following Nrf2 knockdown and MPP^+^ exposure (Figure 2I). Nrf2 SiRNA transfection in SH-SY5Y cells increased their vulnerability to MPP^+^ neurotoxicity as compared to the control SiRNA transfection group (Figure 2I, *p* < 0.05). These data reveal that the protective effect of hydralazine on SH-SY5Y cells exposed to MPP^+^ neurotoxicity is dependent on the Nrf2 pathway.

### Hydralazine Confers Protection in Dopaminergic Neurons in the MPTP Model of Parkinson's Disease

**Figure 3:**
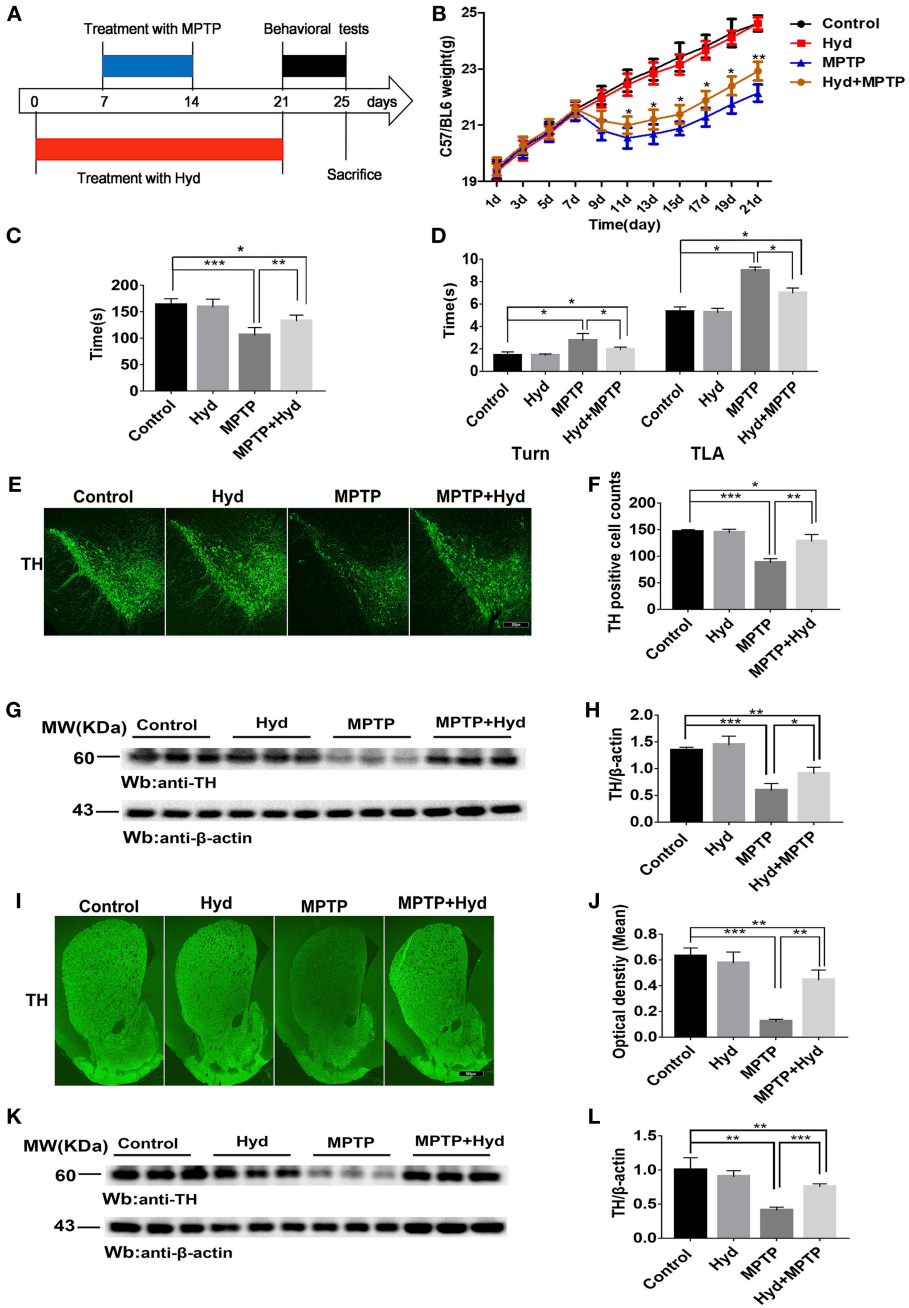
Hydralazine confers protection in dopaminergic neurons in the MPTP model of Parkinson's disease. Schematic representation of the MPTP model experimental design **(A**). Hydralazine alleviated MPTP-induced weight loss **(B)**. ^*^p < 0.05, significant difference between the two indicated groups (*n* = 8). Hydralazine ameliorated MPTP-induced behavior disorder **(C,D)**. Performance on the rotarod and pole test was impaired in MPTP-treated group. However, impairment was ameliorated in MPTP model treated with hydralazine. ^*^p < 0.05, ^**^p < 0.01, ^**^^*^p < 0.001, significant difference between the two indicated groups (*n* = 8). Tyrosine hydroxylase immunofluorescence of substantia nigra pars compacta (SNpc). Scale bar = 250 μm. We found that there were fewer TH positive neurons in the similar anatomic level sections of SN in the MPTP mice than hydralazine- treated mice, MPTP mice with hydralazine and control mice, compared to the MPTP mice group, MPTP mice with hydralazine group significantly attenuated the loss of dopaminergic neurons in SNpc **(E,F)**. ^*^p < 0.05, ^**^p < 0.01, ^***^p < 0.001, *n* = 6, very significant difference between the two indicated groups. The western blotting of TH in SN further reconfirmed the findings (G and H). ^*^p < 0.05, ^***^p < 0.001 *n* = 6, significant difference between the two indicated groups. The MPTP neurotoxicity also resulted the decrease of the TH positive DA terminals in the striatal region of the MPTP mice group compared with MPTP mice with hydralazine group. **(I,J)**. Scale bar = 500 μm. ^**^p < 0.01, ^***^p < 0.001, *n* = 6, very significant difference between the two indicated groups. Immunoblotting also validated this observation **(K,L)**. ^**^p < 0.01, ^***^p < 0.001, *n* = 6, compared with the two indicated groups. Data were presented as mean ± SEM.

To explore the therapeutic effect of hydralazine in PD, the MPTP mice model was used. Mice were divided into four groups (*n* = 9 for per group) for this experiment. The first group mice (MPTP group) only received injections of MPTP-HCl (30 mg/kg, i.p., Sigma) in saline for consecutive 7 days, an MPTP model of PD was generated as previously described (32). The second group of mice (Hyd+MPTP group) were administered with hydralazine (51.7 mg/kg per day in saline, Sigma) (33) by oral gavage for 3 weeks before, during, and after MPTP administration. The third group mice (Hyd group) were administered with hydralazine (51.7 mg/kg per day in saline, Sigma) (33) by oral gavage for 3 weeks, and the fourth group mice (Control group) received vehicle only. The pretreatment paradigm in the MPTP-induced PD Model was utilized (Figure 3A). We observed that the weight of MPTP-treated mice was much lower compared to normal group due to the neurotoxicity of MPTP. But administration of hydralazine alleviated the loss of weight (Figure 3B). Behavior testing of the animals was performed after the last oral gavage (Figure 3A). The rotarod test was applied to evaluate motor and coordination abilities. In this test, the decrease in latent time on the rod in the MPTP mice compared to the vehicle treated control mice (*p* < 0.001) was reversed by hydralazine treatment (*p* < 0.01) (Figure 3C). Similar results were obtained in Pole test. The time to orient downward (Turn) and to descend (TLA) was increase in the MPTP mice compared to the control mice (*p* < 0.05), indicating a motor deficit, and this effect was reversed by hydralazine (*p* < 0.05) (Figure 3D). These results suggested that hydralazine conferred protection in locomotion function.

After the behavioral tests, we tested whether *in vivo* hydralazine can maintain the integrity of dopaminergic neurons in the SNpc and their terminal fibers in striatum. Thus, we performed immunofluorescence staining using dopaminergic neuronal specific marker, anti-TH antibody. MPTP administration caused remarkable loss of dopaminergic neurons in the SNpc (Figures 3E,F) and striatal DA terminal fibers (Figures 3I,J) when compared to control. Meanwhile, we found that reduction of TH-positive neurons was abolished in the similar anatomic level sections of SN in the MPTP mice treated with hydralazine than MPTP alone (Figure 3E). Quantification analysis using the cell counting kit in a double blind way indicated a statistically significant preservation of TH positive dopaminergic SN neurons in the MPTP-treated mice with hydralazine groups compared with MPTP mice (Figure 3F, *p* < 0.01). This observation was further confirmed by western blotting assay on SN lysates (Figure 3G). The ratio of TH/β-action in the MPTP group was significantly lower than in control group (*p* < 0.001) and MPTP mice with hydralazine groups (Figure 3H, *p* < 0.05). Hydralazine-treated mice in the presence of MPTP showed significant reductions in the loss of the TH positive DA terminals in the striatal region when compared to MPTP alone mice (Figures 3I,J, *p* < 0.01). Consistent with TH-immunofluorescent in the striatal region, western blotting of the striatum lysates rescued the loss of TH positive DA terminals in the hydralazine-treated mice with MPTP compared to MPTP alone mice (Figures 3K,L, *p* < 0.001), Taken together, these findings provide evidence that hydralazine confers protection on nigrostriatal dopaminergic neurons in the subacute model of MPTP neurotoxicity.

### Hydralazine Alleviate Oxidative Stress and Activates Nrf2-Triggered Gene Expression *in vivo*

**Figure 4:**
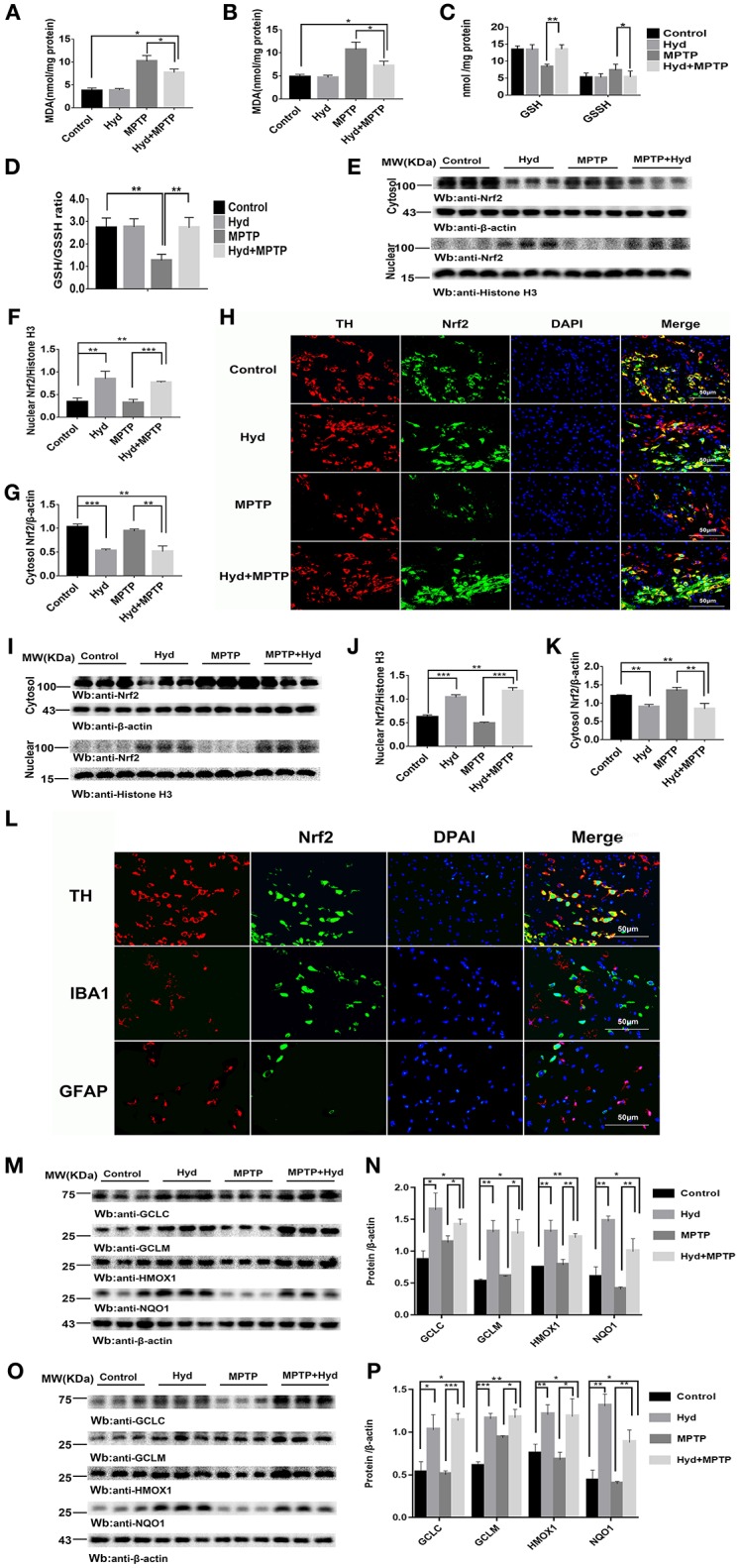
Hydralazine alleviate oxidative stress and activates Nrf2-triggered gene expression *in vivo*. Hydralazine significantly decreased MDA in the substantia nigra (A) and striatum(B) of MPTP mice treated with hydralazine compared with MPTP mice. ^*^p < 0.05, *n* = 5, significant difference between the two indicated groups. MPTP administration showed a remarkable decrease in GSH levels and increase in GSSH that was inhibited by hydralazine in the SNpc, corresponding increase in the ratio of GSH/GSSG **(C,D)**. ^*^p < 0.05, ^**^p < 0.01, *n* = 5, significant difference between the two indicated groups. Hydralazine treatment leads to a shift in Nrf2 migration and increases nuclear Nrf2 Protein levels, in the meantime, cytosolic Nrf2 had corresponding decrease in the substantia nigra **(E–H)** and striatum **(I–K)** of MPTP-treated mice. ^**^p < 0.01, ^***^p < 0.001. *n* = 6, significant difference between the two indicated groups. Nrf2 colocalized with TH in the SNpc, not with IBA1, GFAP by double immunofluorescence **(L)** in the SNpc of hydralazine-treated mice with MPTP, hydralazine can increase nuclear translocation of Nrf2 in the dopaminergic neuron **(L)**. Protein levels of Nrf2 were analyzed by Western blot. β-actin and Histonen H3 are used as markers for cytosolic and nuclear fractions, respectively. Hydralazine treatment increased the expression of Nrf2 downstream target in the substantia nigra **(M,N)** and striatum **(O,P)** of MPTP-treated mice. ^*^p < 0.05, ^**^p < 0.01, ^***^p < 0.001, *n* = 6, significant difference between the two indicated groups. Data were presented as mean ± SEM.

Subsequently, we investigated whether hydralazine activates Nrf2-ARE pathway *in vivo* using mice. Mice were treated as described above to assess the level of MDA, a key product produced from membrane lipid oxidation. Mice treated with MPTP displayed a remarkably increase in MDA levels which were suppressed by hydralazine treatment in the SNpc (Figure 4A) and in striatum (Figure 4B). Glutathione (GSH) is an antioxidant which has the capacity to prevent damage to important cellular components. Glutathione disulfide (GSSG) is its oxidized form and the ratio of oxidized glutathione to reduced glutathione is frequently used to evaluate oxidative stress. MPTP decreased GSH levels and increased GSSH levels and these effects were reversed by hydralazine in the SNpc, as supported by the increased ratio of GSH/GSSG (Figures 4C,D). It can be inferred that Nrf2-ARE signaling suppressed cellular oxidative stress caused by several factors. These experimental results demonstrate that hydralazine suppresses oxidative stress triggered by MPTP.

We further explored whether this effect was due to regulation of the Nrf2-ARE signaling pathway. Figures 4E–G, shows that hydralazine induced a dramatic increase in the nuclear translocation of Nrf2 in the SNpc of hydralazine-treated mice with MPTP, compared with the MPTP mice (*p* < 0.001), corresponding decrease in cytosolic Nrf2 fraction (*p* < 0.01). Moreover, hydralazine treatment alone enhanced the nuclear Nrf2 levels when compared to control (Figures 4E,F, *p* < 0.01). The cytosolic Nrf2 fraction decreased slightly compared to nuclear Nrf2 fraction (Figures 4E,G). This observation was further confirmed by double immunofluorescence (Figure 4H) in the SNpc. Consistent with SNpc, western blotting assay on the striatum lysates showed a statistically significant increase in the Nrf2 nuclear translocation, and a corresponding decrease in cytosolic Nrf2 fraction, in both hydralazine-treated mice with MPTP and hydralazine alone mice when compared to control (Figures 4I,J). Based on these results, it was observed that hydralazine activated Nrf2-ARE signaling. Moreover, we observed that Nrf2 colocalized with TH (dopaminergic neuron marker) in the SNpc, but not with ionized calcium binding adapter molecule 1 (IBA1, microglial cell marker) or glial fibrillary acidic protein (GFAP, astrocyte marker) as revealed by double immunofluorescence in the SNpc of hydralazine-treated mice with MPTP. Hydralazine increased the nuclear translocation of Nrf2 in the dopaminergic neuron (Figure 4L), which explained why hydralazine protected dopaminergic neurons from MPTP induced neurotoxicity to a certain extent.

In the hydralazine treatment group, western blot assay revealed that Nrf2-ARE targeted genes, NQO1, HMOX1, GCLM, and GCLC, were significantly increased in the SNpc (Figures 4M,N) and in striatum (Figures 4O,P) after compare with MPTP alone. Additionally, treatment with hydralazine alone increased the expression of above ARE genes compared to control group in both SNpc (Figures 4M,N) and striatum (Figures 4O,P). Based on the above discussion, the mechanism by which hydralazine activated Nrf2-ARE signaling even in the absence of toxicant was not clear. This should be explored in further studies. In conclusion, these data provide *in vivo* evidence that hydralazine can activate the Nrf2-mediated ARE gene transcription which provides antioxidant effects *in vivo*.

## Discussion

Current treatment for PD are primarily designed relief some of the clinical symptoms which hardly translated to improved quality of life in most patients. In addition, there is no known therapy that prevents the pathogenesis and progress of neurodegeneration. Although several studies have been performed to assess the etiology of PD, the associated mechanisms are still not fully understood. Mounting evidence suggests that oxidative stress appears to be involved in the pathogenesis of PD (36). Consequently, we have reasons to believe that increasing the capacity of dopaminergic neurons to confront oxidants could serve as an important strategy to prevent the onset and/or to delay the progression of PD. However, the prevailing knowledge from clinical trials on antioxidants such as ubiquinone, glutathione, N-acetylcysteine, vitamin C, and vitamin E reveals there are conflicting outcomes on their efficacy (22–25). It is likely that oxidants may exert influence by activating death-related pathways rather than directly killing dopaminergic neurons (37), relying only on stoichiometric scavenging of oxidants has proved to be ineffective. The Nrf2 signaling cascade modulates transcriptional expression of oxidative stress to restore redox homeostasis and is a versatile pathway for neurotherapeutics. Several genetic studies have demonstrated that a functional haplotype in the Nrf2 gene promoter, which conferred high transcriptional activity had a protective effect (38), and the deficiency of Nrf2 increased hypersensitivity of the dopaminergic neurons to neurotoxicity caused by MPTP (14, 39, 40) and 6-OHDA (15) in animal models. Moreover, *in vitro* Nrf2 activation also provides neuroprotective effect against the neurotoxins paraquat (41), 6-OHDA (42, 43), and MPP^+^ (44). These datasets imply that the Nrf2 pathway may provide neuroprotection which can be exploited to develop drugs that treat PD.

Hydralazine, which was developed by Swiss researchers in the early 1950s (45), was approved drug by FDA for antihypertensive treatment. In this study, we demonstrate that hydralazine displays remarkable neuroprotective effects in a model of PD *in vivo* and *in vitro*. Oral administration of hydralazine decreased MDA levels, increased the ratio of GSH/GSSG, alleviated weight loss and the associated motor deficits, attenuated dopaminergic neurons loss and activated Nrf2 signaling pathways, all of which exerted profound neuroprotective activities in MPTP model of PD (Figure 3), and these effects were further confirmed in human neuroblastoma SH-SY5Y cells (Figure 1). The cell viability was increased following hydralazine treatment when exposed to MPP^+^ or H_2_O_2_. Among the key Nrf2-regulated genes stimulated by hydralazine were NQO1, HMOX1, GCLM, GCLC. It was reported NQO1 ameliorated the baleful effects of DA quinines, and had various antioxidant properties (46, 47). HMOX1 has been implicated in the metabolism of the pro-oxidant heme to form the antioxidant pigment biliverdin which confers resistance to chronic oxidative stress and apoptosis in dopaminergic neurons (48, 49). GCLC and GCLM regulates the production of cellular antioxidant glutathione (50). These proteins are involved in oxidative stress and redox homeostasis. Nrf2 SiRNA transfection was performed to explore the function of Nrf2 in the protective effect of hydralazine against neurotoxicity MPP^+^ induced in SHSY5Y cells. Nrf2 SiRNA2 transfection treatment abolished the hydralazine-induced ARE genes expression and increased the vulnerability to MPP^+^ neurotoxicity (Figure 2). However, we lacked Nrf2 knockout mice and Nrf2 knockin mutant mice to perform more confirmatory experiments.

A noteworthy phenomenon is that hydralazine treatment alone increased nuclear translocation of Nrf2 and elevated the Nrf2-mediated ARE gene transcription *in vitro* and *in vivo* (Figures 1, 2, 4). But the mechanism for this effect remains elusive. It is possible that Nrf2 was modified by hydralazine in a way that disrupted the Nrf2:Keap1 interaction, which then allowed it to translocate to the nucleus where it binds the antioxidant response elements (AREs), a consensus gene sequence present in the promoter region of a large number of genes encoding antioxidant enzymes (11). In the future, we will further explore this concept. In addition, we found that hydralazine increased the migration of Nrf2 to the nucleus in dopaminergic neurons, induced the expression of its downstream antioxidative genes, although we did not observe this phenomenon in microglial cells and astrocytes. Collectively, our experimental results demonstrate the ability of hydralazine to modulate key antioxidant genes which are crucial to the survival of SH-SY5Y cells and dopaminergic neurons in PD model.

Hydralazine has been known to act as an antioxidant which eliminates acrolein, an agent that transduces oxidative stress signals (51, 52) and a potent Nrf2 activator (30). Here, we demonstrate that Nrf2 activation is a novel mechanism by which hydralazine exerts protection in PD. However, additional investigations are required to explain how this nucleophilic drug activates the Nrf2 pathway. One possibility is that hydralazine directly disrupts the interaction between Keap-1 and Nrf2 (53). It is also likely that hydralazine activates Nrf2 pathway through other pathways e.g., Notch or AMP kinase pathways, nuclear factor-kappa B (NF-κB), synoviolin, the GSK-3-β-TrCP, and PI3K/Akt pathway (54), which are reported to interact with Nrf2 elements. Another possibility is that the “adduct-trapping” properties of hydralazine which involves the classic electrophile-mediated pathway to increase the degradation of Keap1, thus accelerate Nrf2 liberation (55). Certainly, there is little evidence that hydrazaline crosses the Blood-Brain Barrier, However, this drug has been studied in the Alzheimer's disease mouse model (33). Together with the findings obtained in this study, we speculate that hydrazaline has some degree of permeability through the Blood-Brain Barrier. To improve its clinical application, its oral bioavailability, *in vivo* pharmacokinetic profiles, permeability through Blood-Brain Barrier and the potential side effects need to be explored.

## Conclusion

This study shows that hydralazine as a potent Nrf2 activator, increases the expression of Nrf2 downstream ARE target genes and confers strong neuroprotection in model of PD both *in vitro* and *vivo* in a Nrf2 -independent manner. These datasets provide novel insights that have high therapeutic value for Parkinson's disease.

## Ethics Statement

All animal procedures were in strict accordance with the National Institutes of Health's Guidelines for Care and were approved by the Animal Care and Use Committees of Huazhong University of Science and Technology (HUST).

## Author Contributions

XG participated in all aspects of the experimental design, implementation, analysis, and writing including obtaining, analyzing, and interpreting data and making significant contributions to the writing of the manuscript. CH contributed to the experimental design. KM, YX, FW, SY, LK, YS, JW, and JuH contributed to the experimental implementation. JiH and NX contributed to data analysis. The whole experiment was completed under the guidance of TW.

### Conflict of Interest Statement

The authors declare that the research was conducted in the absence of any commercial or financial relationships that could be construed as a potential conflict of interest.

## References

[1] Bokov A Chaudhuri A Richardson A. The role of oxidative damage and stress in aging. Mech Ageing Dev. (2004) 125:811–26. 10.1016/j.mad.2004.07.009 15541775

[2] Dias V Junn E Mouradian MM. The role of oxidative stress in Parkinson's disease. J Parkinsons Dis. (2013) 3:461–91. 10.3233/JPD-130230 24252804 PMC4135313

[3] Goswami SK Maulik N Das DK. Ischemia-reperfusion and cardioprotection: a delicate balance between reactive oxygen species generation and redox homeostasis. Ann Med. (2007) 39:275–89. 10.1080/07853890701374677 17558599

[4] Kaspar JW Niture SK Jaiswal AK. Nrf2:INrf2 (Keap1) signaling in oxidative stress. Free Radic Biol Med. (2009) 47:1304–9. 10.1016/j.freeradbiomed.2009.07.035 19666107 PMC2763938

[5] Lu SC. Regulation of glutathione synthesis. Mol Aspects Med. (2009) 30:42–59. 10.1016/j.mam.2008.05.005 18601945 PMC2704241

[6] Sheehan D Meade G Foley VM Dowd CA. Structure, function and evolution of glutathione transferases: implications for classification of non-mammalian members of an ancient enzyme superfamily. Biochem J. (2001) 360:1–16. 10.1042/bj3600001 11695986 PMC1222196

[7] Itoh K Wakabayashi N Katoh Y Ishii T Igarashi K Engel JD. Keap1 represses nuclear activation of antioxidant responsive elements by Nrf2 through binding to the amino-terminal Neh2 domain. Genes Dev. (1999) 13:76–86. 10.1101/gad.13.1.76 9887101 PMC316370

[8] Taguchi K Motohashi H Yamamoto M. Molecular mechanisms of the Keap1-Nrf2 pathway in stress response and cancer evolution. Genes Cells. (2011) 16:123–40. 10.1111/j.1365-2443.2010.01473.x 21251164

[9] Kensler TW Wakabayashi N Biswal S. Cell survival responses to environmental stresses via the Keap1-Nrf2-ARE pathway. Annu Rev Pharmacol Toxicol. (2007) 47:89–116. 10.1146/annurev.pharmtox.46.120604.141046 16968214

[10] Kumar H Kim IS More SV Kim BW Choi DK. Natural product-derived pharmacological modulators of Nrf2/ARE pathway for chronic diseases. Nat Prod Rep. (2013) 31:109–39. 10.1039/C3NP70065H 24292194

[11] Sykiotis GP Bohmann D. Stress-activated cap'n'collar transcription factors in aging and human disease. Sci Signal. (2011) 3:re3. 10.1126/scisignal.3112re3 20215646 PMC2991085

[12] Cuadrado A Morenomurciano P Pedrazachaverri J The transcription factor Nrf2 as a new therapeutic target in Parkinson's disease, Expert Opinion on Therapeutic Targets, Informa Healthcare. Expert Opin Ther Targets. (2009) 13:319–29. 10.1517/13543780802716501 19236154

[13] Williamson TP Johnson DA Johnson JA. Activation of the Nrf2-ARE pathway by siRNA knockdown of Keap1 reduces oxidative stress and provides partial protection from MPTP-mediated neurotoxicity. Neurotoxicology. (2012) 33:272–9. 10.1016/j.neuro.2012.01.015 22342405 PMC3521526

[14] Burton N Kensler TR. *In vivo* modulation of the Parkinsonian phenotype by Nrf2. Neurotoxicology. (2006) 27:1094–100. 10.1016/j.neuro.2006.07.019 16959318

[15] Jakel RJ Townsend JA Kraft AD Johnson JA. Nrf2-mediated protection against 6-hydroxydopamine. Brain Res. (2007) 1144:192–201. 10.1016/j.brainres.2007.01.131 17336276 PMC2062573

[16] Rojo AI Innamorato NG Martín-Moreno AM De Ceballos ML Yamamoto M Cuadrado A. Nrf2 regulates microglial dynamics and neuroinflammation in experimental Parkinson's disease. Glia. (2010) 58:588–98. 10.1002/glia.20947 19908287

[17] Lee JA Son HJ Kim JH Park KD Shin N Kim HR. A novel synthetic isothiocyanate ITC-57 displays antioxidant, anti-inflammatory, and neuroprotective properties in a mouse Parkinson's disease model. Free Radic Res. (2016) 50:1188–99. 10.1080/10715762.2016.1223293 27598306

[18] Lee JA Kim JH Woo SY Son HJ Han SH Jang BK. A novel compound VSC2 has anti-inflammatory and antioxidant properties in microglia and in Parkinson's disease animal model. Br J Pharmacol. (2015) 172:1087–100. 10.1111/bph.12973 25297649 PMC4314197

[19] Lee JA Son HJ Park KD Han SH Shin N Kim JH. A novel compound ITC-3 activates the Nrf2 signaling and provides neuroprotection in Parkinson's disease models. Neurotox Res. (2015) 28:1–14. 10.1007/s12640-015-9550-z 26233727

[20] Skibinski G Hwang V Ando DM Daub A Lee AK Ravisankar A From the cover: Nrf2 mitigates LRRK2- and Î±-synucleinâ“induced neurodegeneration by modulating proteostasis. Proc Natl Acad Sci USA. (2017) 114:1165 10.1073/pnas.1522872114 28028237 PMC5293055

[21] Son HJ Choi JH Lee JA Kim DJ Shin KJ Hwang O. Induction of NQO1 and neuroprotection by a novel compound KMS04014 in Parkinson's disease models. J Mol Neurosci. (2015) 56:263–72. 10.1007/s12031-015-0516-7 25702135

[22] Kamat CD Gadal S Mhatre M Williamson KS Pye QN Hensley K. Antioxidants in central nervous system diseases: preclinical promise and translational challenges. J Alzheimers Dis. (2008) 15:473–93. 10.3233/JAD-2008-15314 18997301 PMC2669703

[23] Shen L Ji HF. Insights into the disappointing clinical trials of antioxidants in neurodegenerative diseases. J Alzheimers Dis. (2010) 19:1141–2. 10.3233/JAD-2010-1307 20308779

[24] Shults CW Haas R. Clinical trials of coenzyme Q10 in neurological disorders. Biofactors. (2010) 25:117–26. 10.1002/biof.5520250113 16873936

[25] Storch A Jost WH Vieregge P Spiegel J Greulich W Durner J. Randomized, double-blind, placebo-controlled trial on symptomatic effects of coenzyme Q in Parkinson disease. Arch Neurol. (2007) 64:938–44. 10.1001/archneur.64.7.nct60005 17502459

[26] Burcham PC. Potentialities and pitfalls accompanying chemico-pharmacological strategies against endogenous electrophiles and carbonyl stress. Chem Res Toxicol. (2008) 21:779–86. 10.1021/tx700399q 18275160

[27] Hamann K Nehrt G Ouyang H Duerstock B Shi R. Hydralazine inhibits compression and acrolein-mediated injuries in *ex vivo* spinal cord. J Neurochem. (2010) 104:708–18. 10.1111/j.1471-4159.2007.05002.x 17995940

[28] Hamann K Durkes A Ouyang H Pond A Shi R. Critical role of acrolein in secondary injury following *ex vivo* spinal cord trauma. J Neurochem. (2010) 107:712–21. 10.1111/j.1471-4159.2008.05622.x 18710419 PMC2671023

[29] Liusnyder P Borgens RB Shi R Hydralazine rescues PC12 cells from acrolein-mediated death. J Neurosci. Res. (2010) 84:219–27. 10.1002/jnr.20862 16619236

[30] Dehghan E Zhang Y Saremi B Yadavali S Hakimi A Dehghani M. Hydralazine induces stress resistance and extends C. elegans lifespan by activating the NRF2/SKN-1 signalling pathway. Nat Commun. (2017) 8:2223. 10.1038/s41467-017-02394-3 29263362 PMC5738364

[31] Maheshwari M Roberts JK Desutter B Duong K Tingling J Fawver JN. Hydralazine modifies Aβ fibril formation and prevents modification by lipids *in vitro*. Biochemistry. (2010) 49:10371. 10.1021/bi101249p 21058733 PMC3033120

[32] Jacksonlewis V Przedborski S Protocol for the MPTP mouse model of Parkinson's disease. Nat Protoc. (2007) 2:141–51. 10.1038/nprot.2006.342 17401348

[33] Wang J Zhao Z Lin E Zhao W Qian X Freire D. Unintended effects of cardiovascular drugs on the pathogenesis of Alzheimer's disease. PLoS ONE. (2013) 8:e65232. 10.1371/journal.pone.0065232 23762322 PMC3675203

[34] Yang L Calingasan NY Thomas B Chaturvedi RK Kiaei M Wille EJ. Neuroprotective effects of the triterpenoid, CDDO methyl amide, a potent inducer of Nrf2-mediated transcription. PLoS ONE. (2009) 4:e5757. 10.1371/journal.pone.0005757 19484125 PMC2684590

[35] Burcham PC. Carbonyl scavengers as pharmacotherapies in degenerative disease: hydralazine repurposing and challenges in clinical translation. Biochem Pharmacol. (2018) 154:397–406. 10.1016/j.bcp.2018.06.006 29883705

[36] Jenner P. Oxidative stress in Parkinson's disease. Ann Neurol. (2003) 53 (Suppl. 3):S26. 10.1002/ana.10483 12666096

[37] Zhou C Huang Y Przedborski S. Oxidative stress in Parkinson's Disease: a mechanism of pathogenic and therapeutic significance. Ann N Y Acad Sci. (2008) 1147:93–104. 10.1196/annals.1427.023 19076434 PMC2745097

[38] Otter MV Landgren S Nilsson S Celojevic D Bergström P Håkansson A Association of Nrf2-encoding NFE2L2 haplotypes with Parkinson's disease. BMC Med Genet. (2010) 11:36 10.1186/1471-2350-11-36 20196834 PMC2843602

[39] Chen PC Vargas MR Pani AK Smeyne RJ Johnson DA Kan YW. Nrf2-mediated neuroprotection in the MPTP mouse model of Parkinson's disease: Critical role for the astrocyte. Proc Natl Acad Sci USA. (2009) 106:2933–8. 10.1073/pnas.0813361106 19196989 PMC2650368

[40] Kaidery NA Banerjee R Yang L Smirnova NA Hushpulian DM Liby KT. Targeting Nrf2-Mediated gene transcription by extremely potent synthetic triterpenoids attenuate dopaminergic neurotoxicity in the MPTP mouse model of Parkinson's disease. Antioxid Redox Signal. (2013) 18:139–57. 10.1089/ars.2011.4491 22746536 PMC3514006

[41] Nisosantano M Gonzálezpolo RA BravoSan Pedro JM Gómezsánchez R Lastresbecker I Ortizortiz MA Activation of apoptosis signal-regulating kinase 1 is a key factor in paraquat-induced cell death: modulation by the Nrf2/Trx axis. Free Radic Biol Med. (2010) 48:1370–81. 10.1016/j.freeradbiomed.2010.02.024 20202476

[42] Hara H Ohta M Adachi T. Apomorphine protects against 6-hydroxydopamine-induced neuronal cell death through activation of the Nrf2-ARE pathway. J Neurosci Res. (2010) 84:860–6. 10.1002/jnr.20974 16802348

[43] Hwang YP Jeong HG. The coffee diterpene kahweol induces heme oxygenase-1 via the PI3K and p38/Nrf2 pathway to protect human dopaminergic neurons from 6-hydroxydopamine-derived oxidative stress. Febs Lett. (2008) 582:2655–62. 10.1016/j.febslet.2008.06.045 18593583

[44] Wruck CJ Claussen M Fuhrmann G Römer L Schulz A Pufe T Luteolin protects rat PC 12 and C6 cells against MPP+ induced toxicity via an ERK dependent Keapl-Nrf2-ARE pathway. J Neural Transm Suppl. (2007) 72:57–67. 10.1007/978-3-211-73574-9_9 17982879

[45] Sneader W Drug Discovery: A History. Chichester: John Wiley & Sons Ltd. (2005). 10.1002/0470015535

[46] Kapinya KJ Harms U Harms C Blei K Katchanov J Dirnagl U. Role of NAD(P)H:quinone oxidoreductase in the progression of neuronal cell death *in vitro* and following cerebral ischaemia *in vivo*. J Neurochem. (2010) 84:1028–39. 10.1046/j.1471-4159.2003.01601.x 12603827

[47] Van FM Kuiperij HB The Nrf2-ARE Signalling pathway: promising drug target to combat oxidative stress in neurodegenerative disorders. Curr Drug Targets CNS Neurol Disord. (2005) 4:267–81. 10.2174/1568007054038238 15975029

[48] Ferris CD Jaffrey SR Sawa A Takahashi M Brady SD Barrow RK. Haem oxygenase-1 prevents cell death by regulating cellular iron. Nat Cell Biol. (1999) 1:152–7. 10.1038/11072 10559901

[49] Otterbein LE Soares MP Yamashita K Bach FH. Heme oxygenase-1: unleashing the protective properties of heme. Trends Immunol. (2003) 24:449–55. 10.1016/S1471-4906(03)00181-9 12909459

[50] Franklin CC Backos DS Mohar I White CC Forman HJ Kavanagh TJ. Structure, function, and post-translational regulation of the catalytic and modifier subunits of glutamate cysteine ligase. Mol Aspects Med. (2009) 30:86–98. 10.1016/j.mam.2008.08.009 18812186 PMC2714364

[51] Hamann K Shi R. Acrolein scavenging: a potential novel mechanism of attenuating oxidative stress following spinal cord injury. J Neurochem. (2010) 111:1348–56. 10.1111/j.1471-4159.2009.06395.x 19780896

[52] Luo J Shi R. Acrolein induces oxidative stress in brain mitochondria. Neurochem Int. (2005) 46:243–52. 10.1016/j.neuint.2004.09.001 15670641

[53] Marcotte D Zeng W Hus JC Mckenzie A Hession C Jin P. Small molecules inhibit the interaction of Nrf2 and the Keap1 Kelch domain through a non-covalent mechanism. Bioorg Med Chem. (2013) 21:4011–9. 10.1016/j.bmc.2013.04.019 23647822

[54] O'Connell MA Hayes JD. The Keap1/Nrf2 pathway in health and disease: from the bench to the clinic. Biochem Soc Trans. (2015) 43:687–9. 10.1042/BST20150069 26551713

[55] Ma Q. Role of Nrf2 in oxidative stress and toxicity. Annu Rev Pharmacol Toxicol. (2013) 53:401–26. 10.1146/annurev-pharmtox-011112-140320 23294312 PMC4680839

